# High Performance On-Chip Energy Storage Capacitors with Plasma-Enhanced Atomic Layer-Deposited Hf_0.5_Zr_0.5_O_2_/Al-Doped Hf_0.25_Zr_0.75_O_2_ Nanofilms as Dielectrics

**DOI:** 10.3390/nano13111765

**Published:** 2023-05-30

**Authors:** Yuli He, Guang Zheng, Bao Zhu, Xiaohan Wu, Wen-Jun Liu, David Wei Zhang, Shi-Jin Ding

**Affiliations:** 1 School of Microelectronics, Fudan University, Shanghai 200433, China; 2 Jiashan Fudan Institute, Jiaxing 314100, China

**Keywords:** Al-doped Hf_0.25_Zr_0.75_O_2_, antiferroelectric, energy storage density, energy storage efficiency

## Abstract

Concurrently achieving high energy storage density (ESD) and efficiency has always been a big challenge for electrostatic energy storage capacitors. In this study, we successfully fabricate high-performance energy storage capacitors by using antiferroelectric (AFE) Al-doped Hf_0.25_Zr_0.75_O_2_ (HfZrO:Al) dielectrics together with an ultrathin (1 nm) Hf_0.5_Zr_0.5_O_2_ underlying layer. By optimizing the Al concentration in the AFE layer with the help of accurate controllability of the atomic layer deposition technique, an ultrahigh ESD of 81.4 J cm^−3^ and a perfect energy storage efficiency (ESE) of 82.9% are simultaneously achieved for the first time in the case of the Al/(Hf + Zr) ratio of 1/16. Meanwhile, both the ESD and ESE exhibit excellent electric field cycling endurance within 10^9^ cycles under 5~5.5 MV cm^−1^, and robust thermal stability up to 200 °C. Thus, the fabricated capacitor is very promising for on-chip energy storage applications due to favorable integratability with the standard complementary metal–oxide–semiconductor (CMOS) process.

## 1. Introduction

With the increasing demands for implantable, wearable, portable electronics and Internet of Things (IoTs), miniature energy storage capacitors are essential for self-powered systems and instantaneous high-power output applications through monolithic three-dimensional (3D) integration with the back-end-of-line (BEOL) of integrated circuits, or system-in-package (SiP). Compared with electrochemical capacitors and micro-batteries, solid-state electrostatic capacitors can offer additional advantages, including a smaller volume, a higher power density and more charge/discharge cycles, especially under various extreme conditions, which makes them more suitable for miniaturized energy autonomous systems [1,2,3]. So far, various dielectric materials such as inorganic perovskites, polymers and glass have been studied for electrostatic capacitors, but these materials encounter severe challenges for on-chip or 3D integrated capacitors due to their complex fabrication processes and poor compatibility with standard complementary metal–oxide–semiconductor (CMOS) technology [4].

In recent years, various element-doped HfO_2_ antiferroelectric (AFE) thin films such as Hf*_x_*Zr_1−*x*_O_2_ (0.1 ≤ *x* ≤ 0.4), HfAlO*_x_* and HfSiO*_x_* have been explored for the usage of on-chip electrostatic capacitors, showing high energy storage density (ESD) comparable or even superior to conventional perovskite materials [5,6,7]. Meanwhile, the doped HfO_2_ thin films can be grown via mature atomic layer deposition (ALD) processes at low temperatures of <350 °C. This guarantees its process compatibility with the BEOL of CMOS technology and processibility over 3D structures. Further, in combination with good reliability, excellent thermal stability and eco-friendly properties, the doped HfO_2_ AFE dielectrics are considered to be very promising materials for on-chip energy storage applications [8]. To improve the ESD of capacitors, our recent studies indicate that the employment of a 1 nm Hf_0.5_Zr_0.5_O_2_ underlying layer between the bottom electrode and the AFE Hf_0.25_Zr_0.75_O_2_ layer can promote the generation of the AFE tetragonal (T)-phase in the AFE layer [9]. In addition, Yang et al. reported a HfO_2_/ZrO_2_ nanolaminate with a 2.2 nm HfO_2_ thin film as the insertion layer between the TiN bottom electrode and ZrO_2_ AFE thin film and achieved an ESD of up to 49.9 J cm^−3^ [10]. Therefore, the introduction of a dielectric layer between the AFE layer and the metal electrode is an effective way to increase the ESD. However, an undesired compromise between the ESD and energy storage efficiency (ESE) usually exists for most doped HfO_2_ AFE capacitors [8,11,12]. For example, the HfAlO*_x_* AFE thin film demonstrated a maximal ESD of 63.7 J cm^−3^, with an ordinary ESE of 64.2% at 6.5 MV cm^−1^ [6]. Moreover, an ESE as high as 93% was achieved for the Al:Hf_0.5_Zr_0.5_O_2_ thin film, but the ESD was only 22 J cm^−3^ [13]. Thus, it has always been an ambitious goal to achieve doped HfO_2_ AFE capacitors that simultaneously possess a high ESD and a high ESE. To enhance the ESE of the capacitors, it is reported that the stability of the T-phase can be improved by decreasing the free energy of the T-phase by reducing the deposition temperature of the Hf*_x_*Zr_1−*x*_O_2_ thin film to 210 °C [11]. Moreover, the trace Al doping in HfO_2_ thin films can improve the AFE behavior because of the enhanced electric field stability of the T-phase [13]. In addition, Das et al. demonstrated an effective way to enrich the energy storage performance of single layer Hf*_x_*Zr_1−*x*_O_2_ capacitors by inserting an Al_2_O_3_ interlayer in the middle of the Hf*_x_*Zr_1−*x*_O_2_ thin films [14]. However, there is still a relatively big room for improvement in the energy storage performance of doped HfO_2_ AFE capacitors. 

In this work, by doping the Al element in the Hf_0.25_Zr_0.75_O_2_ AFE layer accompanied by a 1 nm Hf_0.5_Zr_0.5_O_2_ underlying layer, an ultra-high ESD of 81.4 J cm^−3^ and a perfect ESE of 82.9% were achieved simultaneously, accompanied by a robust thermal stability and excellent electric field cycling endurance.

## 2. Materials and Methods

**Figure 1:**
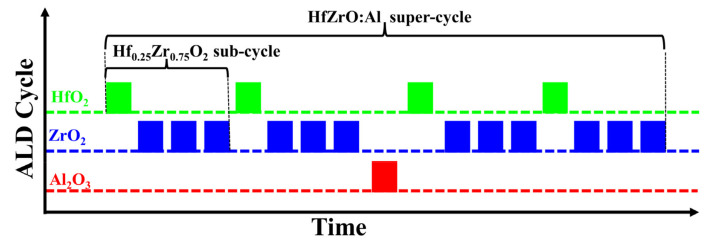
Schematic ALD process steps for the deposition of HfZrO:Al nanofilms with Al/(Hf + Zr) = 1/16.

Capacitors consisting of W/Hf_0.5_Zr_0.5_O_2_/Al-doped Hf_0.25_Zr_0.75_O_2_ (HfZrO:Al)/W were fabricated on 300 nm SiO_2_/Si substrates. The W top and bottom electrodes were deposited via direct current (DC) magnetron sputtering (ACS-4000-C4, ULVAC, Chigasaki, Japan) with a 100 W DC power at room temperature, a high purity tungsten target (>99.95%, ZhongNuo Advanced Material, Beijing, China) and an argon (Ar) gas flow of 20 sccm. The deposited W thin film was about 50 nm. Various nanostacks of 1 nm Hf_0.5_Zr_0.5_O_2_/9 nm HfZrO:Al thin films were deposited via plasma-enhanced ALD (R-200 Advanced, Picosun, Masala, Finland) at 220 °C. The Hf[N(C_2_H_5_)CH_3_]_4_ (TEMAH, >99.99%, J&K Scientific, Beijing, China), Zr[N(CH_3_)_2_]_4_ (TDMAZ, >99.99%, J&K Scientific) and Al(CH_3_)_3_ (TMA, >99.99%, J&K Scientific) were used as the precursors of Hf, Zr and Al, which were kept at 100 °C, 80 °C and 20 °C, respectively. The O_2_ plasma served as the oxygen source, which was generated by a plasma generator integrated within the ALD equipment under 2500 W. In detail, one ALD cycle of HfO_2_ or ZrO_2_ included a 1.6 s vapor pulse of Hf or Zr precursor via an N_2_ carrier gas (80 sccm) and a 10 s O_2_ plasma pulse (130 sccm). Meanwhile, each pulse was followed by a 10 s N_2_ (150 sccm) purging step to remove the excess precursors and post-reaction byproducts. For one ALD cycle of Al_2_O_3_, a 0.1 s TMA pulse via N_2_ carrier gas (100 sccm) and a 0.1 s H_2_O vapor pulse by means of N_2_ carrier gas (150 sccm) were used. In this case, the growth rates of HfO_2_ and ZrO_2_ were both about 0.81 Å/cycle and the growth rate of Al_2_O_3_ was about 0.83 Å/cycle. The Hf_0.5_Zr_0.5_O_2_ and Hf_0.25_Zr_0.75_O_2_ thin films were deposited by fixing the ALD cycle ratios of HfO_2_/ZrO_2_ at 1:1 and 1:3, respectively. Therefore, according to the growth rates of HfO_2_ and ZrO_2_ thin films, a 1 nm Hf_0.5_Zr_0.5_O_2_ thin film was fabricated by alternate deposition of 1 cycle of HfO_2_ and 1 cycle of ZrO_2_ for 6 times, and a 9 nm Hf_0.25_Zr_0.75_O_2_ thin film was prepared by alternate growth of 1 cycle of HfO_2_ and 3 cycles of ZrO_2_ for 27 times. It deserves to be mentioned that the doping of Al in the AFE layer was controlled by super-cycles consisting of Al_2_O_3_ and Hf_0.25_Zr_0.75_O_2_ ALD sub-cycles, and each cycle of Al_2_O_3_ was sandwiched by several sub-cycles of Hf_0.25_Zr_0.75_O_2_. For example, as shown in Figure 1, a super-cycle for the HfZrO:Al thin film consisted of four sub-cycles of Hf_0.25_Zr_0.75_O_2_ and one cycle of Al_2_O_3_, and the relative content of Al in the AFE layer was defined as the ALD cycle ratio of Al_2_O_3_ to (HfO_2_ + ZrO_2_), abbreviated as Al/(Hf + Zr). Therefore, the Al doping content in this sample was defined as 1/16. The Al doping content in other samples, which varied from 1/40 to 1/12, was also defined like this. The Al_2_O_3_ deposition in each super-cycle was inserted in the middle, except for the sample with the cycle ratio of Al/(Hf + Zr) = 1/12, in which the Al_2_O_3_ was sandwiched between 1 and 2 sub-cycles of Hf_0.25_Zr_0.75_O_2_. Then, different super-cycles were repeated for several times until the HfZrO:Al thin films reached their target thicknesses. The capacitor dimensions (square pattern) with an 80 μm side length and a 100 μm distance were determined via photolithography process using a mask alignment system (NXQ4006, N&Q, Morgan Hill, CA, USA). The exposed Hf_1−*x*_Zr*_x_*O_2_ thin films were etched via reactive ion etching system (RIE, SI500, SENTECH, Berlin, Germany) with boron trichloride (BCl_3_) and Ar mixed gas until the W bottom electrode was exposed. The etching process was carried out for 300 s with a source power of 800 W, and the BCl_3_ and Ar gas flow rates were set to 100 sccm and 20 sccm, respectively. Finally, the fabricated capacitors were annealed via the vacuum tube furnace (SKGL-1200, SIOMM, Shanghai, China) in the forming gas (96% N_2_/4% H_2_) at 450 °C for 30 min in order to generate antiferroelectricity. The capacitors with Al/(Hf + Zr) = 1/16 were annealed in the forming gas (96% N_2_/4% H_2_) at 380~480 °C for 30 min in order to investigate the effect of the annealing temperature on the *P–E* hysteresis loops and energy storage performance.

The crystal structures of the Hf_1−*x*_Zr*_x_*O_2_ thin films were analyzed by a grazing-angle incidence X-ray diffractometer (GIXRD, D8 Advance, Bruker AXS GmbH, Karlsruhe, Germany) with an incidence angle of 0.5°. The measurements of polarization–electric field (*P–E*), current density–electric field (*J–E*), thermal stability and electric field cycling endurance of the devices were carried out using a ferroelectric test system (Precision Premier II, Radiant Technologies, Albuquerque, NM, USA) connected to a heating probe station (Summit12000, FormFactor, Livermore, CA, USA). The experiment of thermal stability was performed by raising the temperature of the probe station from 25 to 225 °C. The endurance characteristics were subjected to bipolar square-wave field pulse of 5~6 MV cm^−1^ at a frequency of 50 kHz, and the reading pulses were subjected to triangular-wave field pulse of 5~6 MV cm^−1^ at a frequency of 10 kHz. The leakage current and breakdown electric field of the samples was measured using a semiconductor device analyzer (B1500A, Keysight, Santa Rosa, CA, USA). The capacitance–voltage (*C–V*) curves of the capacitors were measured using a precision impedance analyzer (4294A, Keysight, Santa Rosa, CA, USA). 

## 3. Results and Discussion

**Figure 2:**
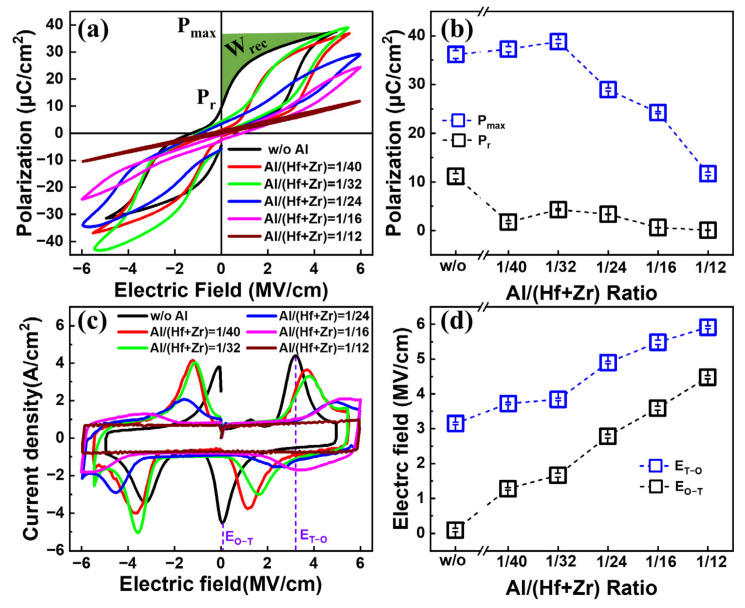
(**a**) Typical *P–E* hysteresis loops and (**b**) the maximum polarization and remanent polarization; (**c**) *J–E* curves and (**d**) the transition electric fields between O- and T-phase of the capacitors with various Al/(Hf + Zr) ratios. Ten devices were measured for each sample.

Figure 2a shows the *P–E* characteristics of the fabricated capacitors with various Al/(Hf + Zr) ratios under a triangle pulse train with a frequency of 10 kHz. By increasing the Al concentration, the maximum polarization (*P*_max_) and remanent polarization (*P*_r_) show a downward trend, accompanied by more and more pinched hysteresis loops. In detail, as shown in Figure 2b, the *P*_max_ value increases slowly from 36.17 to 38.81 μC cm^−2^, and then drops sharply to 11.68 μC cm^−2^. Moreover, the *P*_r_ value decreases from 11.1 to 0.04 μC cm^−2^. This may be due to an increasing concentration of the T-phase in the AFE layer, which can cause a small *P*_r_ value. The corresponding *J–E* curves are shown in Figure 2c. Four discrete peaks located in four different quadrants of the *J–E* curves indicate the AFE properties of the Hf_0.5_Zr_0.5_O_2_/HfZrO:Al nanostacks, and each current peak corresponds to a transition field (E_T-O_) from the T-phase to the orthorhombic (O)-phase, or E_O-T_ from the O-phase to the T-phase. As the Al doping concentration increases, both E_T-O_ and E_O-T_ exhibit an increasing trend. More specifically, the E_T-O_ increases from 3.15 to 5.92 MV cm^−1^ and the E_O-T_ increases from 0.1 to 4.49 MV cm^−1^, as shown in Figure 2d. However, the peak current densities show a decreasing trend. This should be ascribed to the enhanced stability of the T-phase in the Al-doped AFE thin film, leading to weak phase transitions between the T- and O-phases [13].

**Figure 3:**
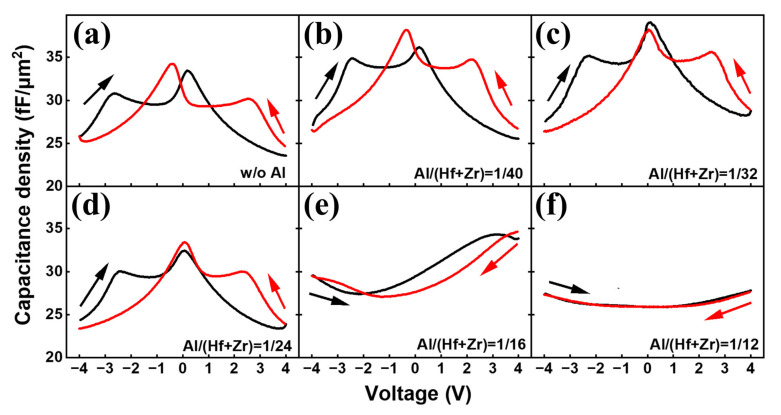
(**a**–**f**) C–V curves of the capacitors with different Al/(Hf + Zr) ratios at 100 kHz.

Figure 3a–f depicts the C–V characteristics of the capacitors with different Al/(Hf + Zr) ratios in order to demonstrate the evolution of the capacitance density in the Hf_0.5_Zr_0.5_O_2_/HfZrO:Al nanostacks enabled by increasing the Al doping concentration. It is found that four discrete capacitance peaks appear for the sample without Al doping, revealing the formation of antiferroelectricity [15]. When the ratio of Al/(Hf + Zr) increases from 1/40 to 1/24, the two capacitance peaks around 0 V tend to merge into a new peak, and the maximum capacitance density is obtained near V = 0, implying an enhancement of the antiferroelectricity [15]. However, by continuously increasing the Al concentration, the capacitance peaks vanish, and finally, the C–V curves become linear dielectric characteristics, which are consistent with the *P–E* curves that become linear when the Al/(Hf + Zr) ratio increases to 1/12. This may be attributed to a change in the crystallization of the HfZrO:Al nanofilms [6]. Therefore, it is clear that the evolution of the capacitance density is achieved by increasing the Al doping concentration.

The ESD of an energy storage capacitor is usually calculated via an integral of the applied electric field (E) with respect to polarization (see a representative case in Figure 2a), which is expressed as follows [16].

$$\mathrm{ESD}=-\int_{P_{\max}}^{P_{r}}\mathrm{EdP}$$

Furthermore, the ESE can be defined as the percentage of the ESD in relation to the total stored energy density, which can be calculated according to the following formula [16]:

$$\mathrm{ESE}=\frac{-\int_{P_{\max}}^{P_{r}}\mathrm{EdP}}{\int_{0}^{P_{\max}}\mathrm{EdP}}\times 100\%$$

**Figure 4:**
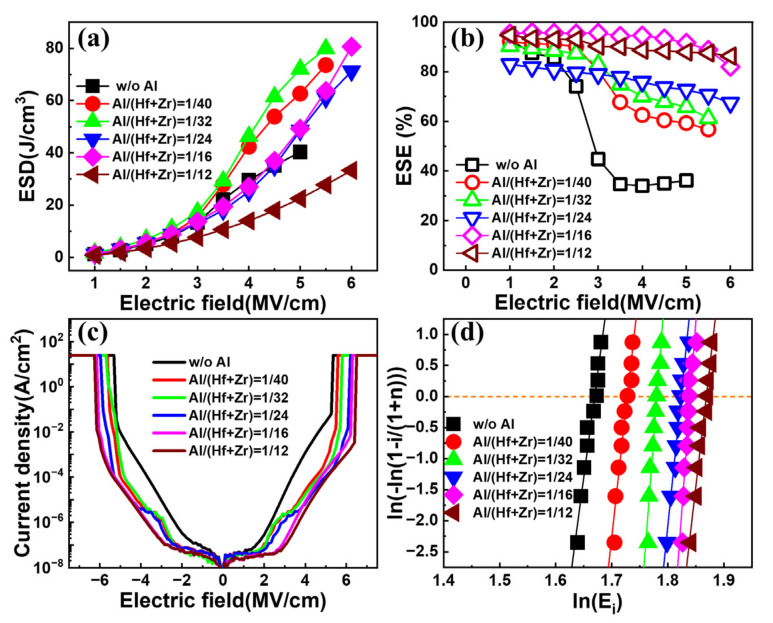
(**a**) The extracted ESD and (**b**) ESE under different electric fields for the capacitors with different Al/(Hf + Zr) ratios; (**c**) the DC current density–electric field curves and (**d**) Weibull distributions of breakdown electric fields of the capacitors with various Al/(Hf + Zr) ratios.

Figure 4a,b show the extracted ESDs and ESEs of the fabricated capacitors with various Al/(Hf + Zr) ratios as a function of the electric field, respectively. All the capacitors exhibit an increasing ESD with the enhancing electric field. In terms of the sample without Al doping, the resulting ESD shows a gradual rise with the electric field until it reaches a near-maximum of ~40.3 J cm^−3^ at 5 MV cm^−1^. However, when the electric field increases slightly, the capacitor encounters an electrical breakdown. For the samples with an Al/(Hf + Zr) ratio range of 1/40~1/16, the resulting maximum ESDs are much higher than that for the undoped sample. However, when the Al/(Hf + Zr) ratio increases to 1/12, the ESD deteriorates badly. As shown in Figure 4b, the corresponding ESE decreases gradually with the increasing electric field for all the capacitors. However, the undoped sample shows a sharp drop in the ESE when the applied electric field exceeds 2 MV cm^−1^, demonstrating a very poor ESE of 36.2% at 5 MV·cm^−1^. Fortunately, Al doping in the AFE layer remarkably improves the ESE, especially under high electric fields. The above results indicate that suitable Al doping in Hf_0.25_Zr_0.75_O_2_ can obviously enhance the breakdown electric field and suppress the energy loss of the capacitor. In order to exclude that the extraction of ESDs under a high electric field are interfered by the leakage current, the I–V characteristics until the electrical breakdown are measured under DC biases. The current density–electric field curves of the capacitors with different Al/(Hf + Zr) ratios are shown in Figure 4c, and the maximum current densities are in the range of 1~20 mA cm^−2^ for the capacitors under the highest *P–E* measurement electric field of 5~6 MV cm^−1^. Even if the above maximum current is flown through the energy storage capacitor during the *P–E* measurement within ten percent of the duration, i.e., ~10 μs, only 0.01~0.2 μC cm^−2^ of the polarization would be overestimated, which is negligible compared with the *P*_max_ of the capacitors. The Weibull distribution of the breakdown electric field (E_B_) of the capacitors with different Al/(Hf + Zr) ratios is shown in Figure 4d. The E_B_ is extracted from the intersection of the fitting line and the X axis when Y = 0. Compared with the sample without Al doping, the Al-doped samples generate higher E_B_. Meanwhile, the resulting E_B_ gradually increases with the increasing Al/(Hf + Zr) ratio of the AFE layer, i.e., from 5.63 to 6.48 MV cm^−1^, which is helpful to improve the ESD.

**Figure 5:**
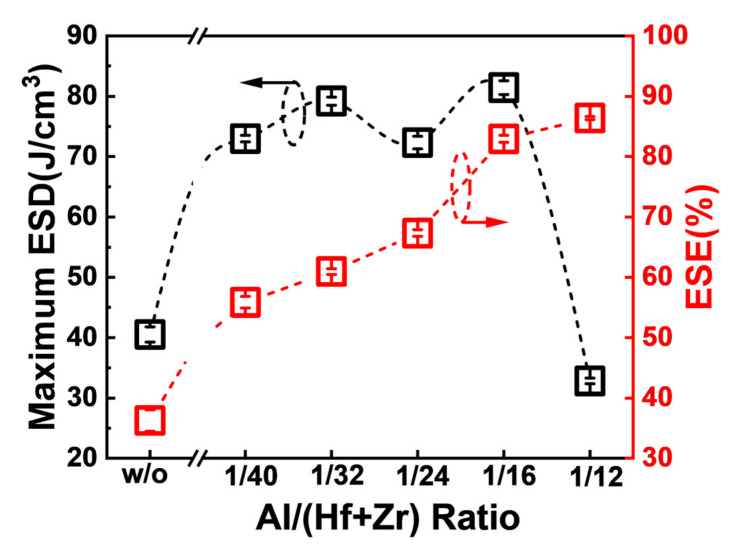
The extracted maximum ESDs and the corresponding ESEs of the capacitors with various Al/(Hf + Zr) ratios. Ten devices were measured for each sample.

Figure 5 shows the maximum ESDs and the corresponding ESEs of the capacitors associated with diverse Al concentrations in the AFE layer. When the ratio of Al/(Hf + Zr) is in the range of 1/40~1/16, the fabricated capacitors exhibit quite high maximum ESDs, and the highest ESD is as large as 81.4 J·cm^−3^ for the Al/(Hf + Zr) ratio of 1/16. Furthermore, the corresponding ESE increases gradually up to 86.4% with increasing the ratio of Al/(Hf + Zr) to 1/12 in the AFE layer. An ideal ESE of 82.9%, corresponding to the largest ESD, is achieved at the Al/(Hf + Zr) ratio of 1/16. 

**Figure 6:**
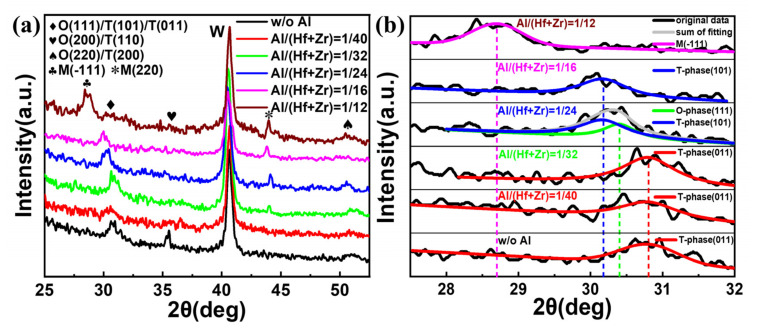
(**a**) GIXRD patterns of the undoped sample and the Hf_0.5_Zr_0.5_O_2_/HfZrO:Al nanostacks with various Al/(Hf + Zr) ratios from 1/40~1/12, (**b**) the de-convolution of the diffraction peak at 2θ ≈ 30° for the undoped sample and the Hf_0.5_Zr_0.5_O_2_/HfZrO:Al nanostacks with various Al/(Hf + Zr) ratios from 1/40~1/12.

To find out the influence of the different Al/(Hf + Zr) ratios on the crystallization characteristics of the investigated dielectrics, the W top electrodes were removed using the RIE etching process after post-annealing at 450 °C for 30 min. Then, the resulting samples with W bottom electrodes and Hf_0.5_Zr_0.5_O_2_/HfZrO:Al nanostacks were analyzed via GIXRD, and the obtained results are shown in Figure 6a. It is clear that all the Hf_0.5_Zr_0.5_O_2_/HfZrO:Al nanostacks with Al/(Hf + Zr) ratios from 1/40 to 1/12 as well as without Al are crystallized well, and the strong peak located at around 30°, resulting from the mixed M-, O- and T-phases in the Hf_0.5_Zr_0.5_O_2_/HfZrO:Al nanostacks, gradually shifts from 30.8° to 28.6°. In addition, three weak diffraction peaks at 35.6°, 44.1° and 51.1° result from O- (200)/T- (110), M- (220) and O- (220)/T- (200) phases, respectively, and the strongest diffraction peak originates from the bottom W thin films [6,17,18]. Moreover, the diffraction peak of O (200)/T (110) disappears and the peak of M (220) appears when the Al element is doped in the AFE layer, and the intensity of the M (220) diffraction peaks gradually increases when the Al/(Hf + Zr) ratio rises from 1/32 to 1/12. Since the diffraction peaks at 2*θ* = ~30° for the Hf_1−*x*_Zr*_x_*O_2_ thin films are superimposed peaks, and the corresponding O- (111), T- (101), (011) and M- (220) phases are very close due to their structural similarities [19,20], the relative phases are also further analyzed to monitor the phase transition, as shown in Figure 6b. Regarding the sample without Al doping, the resulting peak is located at 30.8°, corresponding to the T- (011) phase, and the position of the resulting peaks have no change, as the Al/(Hf + Zr) ratio rises from 1/40 to 1/32. When the Al/(Hf + Zr) ratio is 1/24, the resulting two peaks appear at 30.4° and 30.17°, which are related to the O- (111) and T- (101) phases, respectively. However, when the Al/(Hf + Zr) ratio rises to 1/16, the resulting peak is only located at 30.17°, corresponding to the T- (101) phase. When the Al/(Hf + Zr) ratio further rises to 1/12, both of the O- and T-phase disappear, and the resulting peak is located at 28.6°, corresponding to the M- (-111) phase. The aforesaid results indicate that the doping Al element in the AFE layer prevents the Hf_0.5_Zr_0.5_O_2_/HfZrO:Al nanostacks from forming the O- (200)/T- (110) phase, and generates a new non-ferroelectric M- (220) phase. Moreover, the proportion of the M-phase gradually increases with the increasing Al/(Hf + Zr) ratio. Such an increase in the proportion of the M-phase is unfavorable to the AFE behavior, thus causing the *P*_max_ to decrease with the increasing Al/(Hf + Zr) ratios [12]. In addition, the formation of the extra O- (111) phase may be responsible for the decrease in the ESD in the sample with the Al/(Hf + Zr) ratio of 1/24 due to the reduction in the relative proportion of the T-phase. 

Moreover, Table 1 compares our work with other HfO_2_-based AFE capacitors [5,6,7,9,10,13,14,21,22]. It is observed that our capacitor with the Al/(Hf + Zr) ratio of 1/16 demonstrates the highest ESD at 6 MV cm^−1^ together with a perfect ESE.

**Figure 7:**
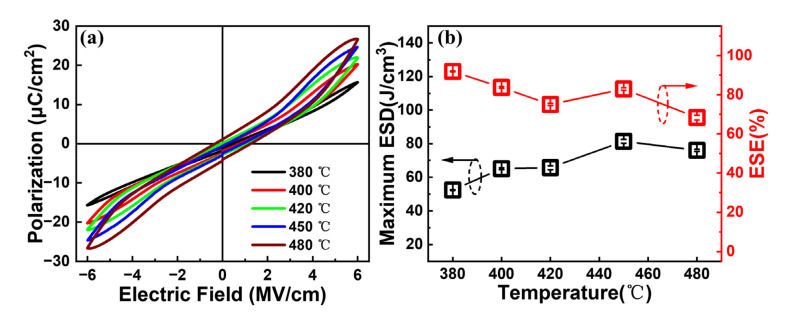
(**a**) Typical *P–E* hysteresis loops and (**b**) the maximum ESD and corresponding ESE of the capacitor with Al/(Hf + Zr) = 1/16 at different annealing temperatures for 30 min. Ten devices were measured for each sample.

In order to investigate the influence of the annealing temperature on the energy storage performance, an additional post-annealing process under various annealing temperatures is carried out for the capacitors with Al/(Hf + Zr) = 1/16. Figure 7a shows the influence of the annealing temperature on the *P–E* hysteresis loops. When the annealing temperature increases from 380 to 480 °C while fixing the annealing time of 30 min, the *P*_max_ value increases from 15.7 to 26.8 μC cm^−2^ and the *P*_r_ value increases from 0.36 to 4 μC cm^−2^, accompanied by more and more outspread hysteresis loops. The maximum ESD and ESE of the capacitors with different annealing temperatures are shown in Figure 7b; the obtained maximum ESD initially increases from 52.4 to 81.4 J cm^−3^, and then decreases to 76.1 J cm^−3^ at the annealing temperature of 480 °C. Additionally, the corresponding ESE demonstrates a decreasing tendency, reaching a value of 82.9% after annealing at 450 °C. These results indicate that 450 °C can be considered as the optimized annealing temperature for the capacitors with Al/(Hf + Zr) = 1/16. It should be noted that when the annealing temperature is ≤420 °C, it is compatible with the BEOL process. Meanwhile, the capacitors with Al/(Hf + Zr) = 1/16 still have excellent energy storage performance, and the maximum ESD and corresponding ESE are 65.7 J cm^−3^ and 75%, respectively.

**Figure 8:**
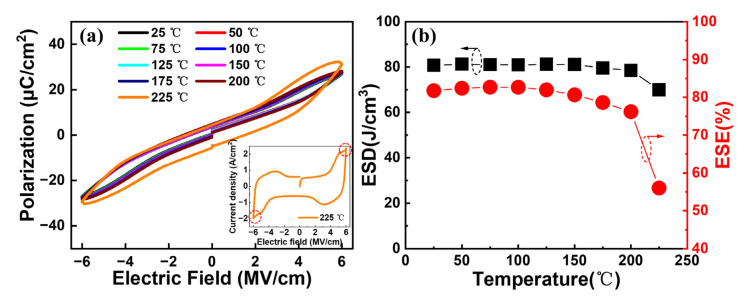
(**a**) Typical *P–E* hysteresis loops and (**b**) ESD and ESE of the capacitors with Al/(Hf + Zr) = 1/16 as a function of measurement temperature.

Further, the temperature reliabilities of our interested capacitors annealed at 450 °C are evaluated. Figure 8a shows the typical *P–E* curves at different temperatures for the Al/(Hf + Zr) ratio of 1/16. A gradual expansion of the *P–E* loops can be observed as the measurement temperature increases from 25 to 225 °C, followed by a sharp expansion at 225 °C. This is because a large leakage current occurs when the temperature increases to 225 °C, as shown in the inset in Figure 8a. Figure 8b shows the extracted ESD and ESE at various temperatures. It is found that the maximum ESD shows a robust thermal stability until 200 °C, with a slight degradation of 2.8%. Meanwhile, the corresponding ESE also reveals a small decrease by 6.8% at 200 °C. However, the ESD and ESE decreased by 14% and 32.4% at 225 °C, respectively, which is attributed to the increased *P*_r_ value and the outspread *P–E* hysteresis loop caused by the increasing leakage current.

**Figure 9:**
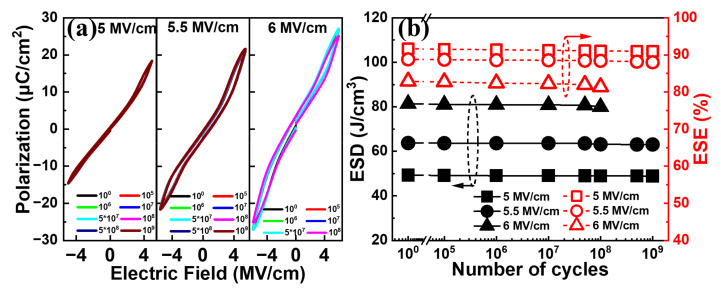
(**a**) Typical *P–E* curves and (**b**) ESD and ESE of the capacitors with Al/(Hf + Zr) = 1/16 at various electric field cycles with a pulse field amplitude of 5~6 MV cm^−1^ at 50 kHz.

Figure 9a shows the typical *P–E* curves for the Al/(Hf + Zr) ratio of 1/16 at various electric field cycles with a pulse field amplitude of 5~6 MV cm^−1^ at 50 kHz. It is observed that no significant change can be seen for the electric field cycle-dependent *P–E* curves until 10^9^ cycles, when the pulse field amplitude is 5 or 5.5 MV cm^−1^. When a higher pulse electric field of 6 MV cm^−1^ is applied, the *P–E* curve is slightly degraded after 10^8^ cycles, and an increased number of pulse cycles may lead to the electrical breakdown of the device. Moreover, as shown in Figure 9b, both the ESD and ESE exhibit negligible degeneration after 10^9^ cycles at 5.5 MV cm^−1^ or 10^8^ cycles even at 6 MV cm^−1^, demonstrating excellent electric field cycling endurance.

## 4. Conclusions

An ultra-high ESD and a perfect ESE are successfully implemented through using 1 nm Hf_0.5_Zr_0.5_O_2_/9 nm HfZrO:Al AFE nanostacks. By optimizing the Al concentrations of the AFE layer, the highest ESD of 81.4 J cm^−3^ is obtained together with a perfect ESE of 82.9% for the Al/(Hf + Zr) ratio of 1/16. This is because the Al doping can enhance the transition electric field between the T- and O-phases and reduce the energy loss of the capacitor. Moreover, the above capacitor also demonstrates a superior thermal stability from 25 to 200 °C, and an excellent electric field cycling endurance of up to 10^9^ cycles at 5~5.5 MV cm^−1^. This makes it more competitive for practical applications. In a word, our work demonstrates an effective means to accomplish high-performance, environmentally friendly and CMOS-compatible energy storage on-chip capacitors.

## Figures and Tables

**Table 1:** Comparison of ESD and ESE between our work and other HfO_2_-based AFE capacitors.

Material	ESD (J cm^−3^)	ESE (%)	Corresponding E (MVcm^−1^)	Ref.
Hf_0.3_Zr_0.7_O_2_	46	50	4.5	[5]
Si:HfO_2_	65	65	4.5	[7]
Al:HfO_2_	42	55	4.5	[21]
Si:Hf_0.5_Zr_0.5_O_2_	50	80	4.5	[13]
La:Hf_0.5_Zr_0.5_O_2_	52	70	4	[22]
Al:Hf_0.5_Zr_0.5_O_2_	22	93	4.5	[13]
HfAlO*_x_*	63.7	64.2	6.5	[6]
HfO_2_/ZrO_2_	49.9	61.9	4.0	[10]
HZO/Al_2_O_3_/HZO *	55	68	4.5	[14]
Hf_0.5_Zr_0.5_O_2_/Hf_0.25_Zr_0.75_O_2_	72	57.8	6	[9]
Hf_0.5_Zr_0.5_O_2_/HfZrO:Al **	81.4	82.9	6	This work

* Hf:Zr = 1:2; ** Al/(Hf + Zr) = 1/16.

## Data Availability

The data presented in this study are available upon request from the corresponding authors.
